# A prospective observational study testing liquid crystal phase change type thermometer placed on skin against oesophageal/pharyngeal placed thermometers in participants undergoing general anesthesia

**DOI:** 10.1186/s12871-019-0881-9

**Published:** 2019-11-09

**Authors:** G. Simpson, R. N. Rodseth

**Affiliations:** 1 Metropolitan Department of Anaesthetics, Critical Care and Pain Management, University of KwaZulu-Natal, 4 Montrose, Hillcrest, Pietermaritzburg, South Africa; 2 Jones, Bhagwan and Partners, Specialist Anaesthetists, Pietermaritzburg, South Africa

**Keywords:** Thermoregulation, Thermometry, Thermometers, Intraoperative monitoring, Observational study

## Abstract

**Background:**

Patient outcomes are influenced by intraoperative temperature management. Oesophageal/pharyngeal temperature monitoring is the standard of care at our institute but is not well tolerated in awake patients. Many non-invasive temperature monitors have been studied. Only the TraxIt® Wearable Children’s Underarm Thermometer which contains liquid crystals that undergo phase changes according to temperature is available at our institution. We tested these non-invasive monitors against our standard of care which is the oesophageal/pharyngeal temperature monitor.

**Methods:**

We conducted a prospective observational study of 100 patients receiving general anaesthesia for elective surgery. Patients were eligible for inclusion if they were ≥ 18 years old, were planned to have a general anaesthetic > 60 min during which no body cavity (chest or abdomen) would be opened. Patient temperature was measured with an oesophageal/pharyngeal thermistor probe and skin surface temperature monitors placed over the forehead, in the axilla, over the sternum, and behind the ear (over major vessels to the brain). Temperatures were recorded and then analysed using Altman-Bland plots. Pre-determined clinically relevant limits of agreement were set at −/+ 0.5 °C.

**Results:**

From the 100 patients we collected 500 data points for each monitor with an average monitoring time of 102 min (30–300 min) across a range of surgical procedures. None of the skin surface temperature monitors achieved the pre-determined limits of agreement and results were impacted by the use of a forced air warmer.

**Conclusion:**

The TraxIt® Wearable Children’s Underarm Thermometers are not suitable for temperature monitoring during general anaesthesia.

## Background

Patient outcome after surgery is strongly influenced by intraoperative temperature management [1–3]. Oesophageal and pharyngeal temperature probes are the standard of care for intraoperative temperature monitoring but are not well tolerated in awake patients [4]. Temperature monitoring in the awake patient is available and range from invasive (e.g. Pulmonary artery catheter thermistor) to semi-invasive (e.g. oral) and non-invasive (e.g. SpotOn Thermometer [5], DoubleSensor thermometer [6] and Temple Touch Pro [7]), however these are not widely endorsed or available for use at our institution.

An alternative temperature monitoring device available at our institution is the Traxit® Wearable Children’s Underarm Thermometer. This thermometer utilises liquid crystals that have phase change properties when heated and is read using a dot matrix grid. The purported advantage of such a monitor is that it could be placed on the patient before anaesthesia while in the ward and could serve as the sole temperature monitor throughout the patient’s anaesthetic journey from ward to theatre and back again.

We tested the performance of these monitors using an oesophageal/pharyngeal temperature monitor as a reference in a prospective observational study in patients undergoing elective surgery.

## Methods

Approval for this study was obtained from the University of KwaZulu-Natal Bio-Ethics Research Committee (BREC BE078/17). Permission was obtained from Grey’s Hospital, Pietermaritzburg, KwaZulu-Natal to recruit participants after approval was granted by the Health Research Committee of the KwaZulu-Natal Department of Health (NHRD Ref: KZ_2016RP_297).

We conducted a prospective observational study in 100 adult patients receiving general anaesthesia for elective surgery. Patients were eligible for inclusion if they were ≥ 18 years old, planned to have an anaesthetic for longer than 60 min (to allow sufficient time for monitor warm up and increase the number of data points for analysis) during which no body cavity would be opened, this was to limit possible confounders such as fluid and heat loss from exposed bowel or possible need for rapid or large volume fluid administration that are common in emergency setting and open body cavity procedures. Patient refusal was the only exclusion criteria.

An oesophageal/pharyngeal thermistor temperature probe was placed between 10 and 20 cm from the nares, as insertion to this depth provides temperatures comparable to distal oesophageal temperatures and acted as a reference temperature [5]. This thermistor probe was not calibrated outside of the mandated yearly maintenance. This thermistor probe is the standard of care for temperature monitoring while under general anaesthesia at our institution (i.e. Is routinely inserted to monitor temperature whilst patients are under general anaesthesia). These measurements were then used to test the performance of the study thermometer – a skin temperature monitor that utilises liquid crystal phase change properties and is read using a dot matrix (TraxIt® Wearable Children’s Underarm Thermometer, already available and in use at our institution). Study thermometers were placed behind the ear (over major vessels to the brain), in the axillary apex (as recommended by manufacturer), over the sternum (over the precordium/heart), and over the middle of the forehead (may represent temperature of underlying brain) and as directed by previously published studies of temperature monitoring.

Temperatures were recorded at 15-min intervals, time 0 being the time when the probe was first placed. The study thermometers do not require calibration before use and have a manufacture reported accuracy of − 0.1 °C and + 0.2 °C within the range of 35.0 °C to 39.0 °C. Forced air warming devices (FAW) (3 M™ *Bair Hugger*™ System) were used at the discretion of the anaesthesia provider based on the oesophageal/pharyngeal temperature monitor. Upper body FAW were used, these cover the upper chest and both arms when a patient is in a supine position. An upper body FAW does cover the skin temperature monitor placed over the sternum.

Using a standardized data collection tool (Additional file 1) we collected patient demographics (age, gender, weight, height), type of surgery, use of a FAW, and the temperature reading on the monitors at 15-min intervals. In addition, the data collection tool provided a comments section to provide subjective feedback from the anaesthetic provider regarding the use of the monitoring devices.

Eligible study participants were approached and verbally informed about the study. If they were interested in participating, they were provided with written study information in English or isiZulu and written consent was obtained. A written copy of the study information and contact details of the principal investigator were given to the participants. Participants were assigned study numbers and their identifiable details were separated from the study information to ensure anonymity. Data was analysed using Microsoft Excel.

Assuming an effect size difference of 0.5, an α of 0.05, and 80% power we required a sample size of 64 participants. However, as suggested by Professor Martin Bland (https://www-users.york.ac.uk/~mb55/meas/sizemeth.htm) [8], we decided to include 100 participants using pragmatic sequential sampling. We conducted 5 assessments, first a composite comparison and then each monitor site’s performance compared to the oesophageal/pharyngeal monitor. We also examined the impact of FAW on the results.

Difference plots, also known as Altman and Bland plots, were used to assess the performance of the skin surface temperature monitor readings as obtained from the four difference sites to the oesophageal/pharyngeal temperature readings. Difference plots aim to compare the test monitoring device to the conventional monitoring device. They plot the difference between the reading of the two monitors against the average of the readings of the two monitors. A bias line is calculated as the average of the difference between the two readings and represents the average discrepancy between the two methods. Upper and lower limits of agreement represent two standard deviations either side of the bias line. However, prior temperature studies have determined that the clinically useful limits of agreement are +/− 0.5 °C [9]. The interpretation of Altman-Bland plots relies on these clinically determined limits of agreement, how wide the limits of agreement are, and whether a trend emerges in the variability of the data points across the plot.

Incomplete and insufficient data points were reported in the results and then excluded from analysis. Participants were examined in the post-anaesthetic care unit to ensure no adverse skin reactions to the skin temperature monitors.

## Results

The study recruited 100 participants from October 2017 to January 2018. All potential participants approached consented to be enrolled in the study. The median age of participants was 39.5 years (range 18–73 years), 53% were female and procedures ranged from general surgery with laparoscopic approach (36%) to burns (2%). Other procedures included orthopaedic upper and lower limb surgery, laparoscopic gynaecological procedures and cystoscopic urological procedures. Participants were under anaesthesia for a median time of 90 min with 60 min being the most frequent duration of anaesthesia (range 30 to 300 min). FAW devices were used for 75% of the participants. Fifty-five of the 400 monitors tested failed to heat up during the recording period: 23% of the forehead monitors, 10% of sternal, 9% of temperature monitor placed behind the ear (over major vessels to the brain), and 13% of axillary monitors. There were 8 incomplete data sheets and 4 participants were not monitored for the correct length of time - these incomplete data points were not included in the analysis.

Interpretation of the Altman Bland plots are included below each plot. The aim of this study was to determine the agreement between the two monitors. If the monitors perfectly agreed all data points would fall on the zero-line indicating no difference in readings. The bias line (i.e. the extent to which one method varies with respect to another when the two methods are compared is plotted as a red solid line) shows the mean difference or average discrepancy between the two monitors. Two standard deviations above and below the bias line represent the upper and lower limits of agreement (yellow broken lines). In this study we also included predetermined limits of agreement of +/− 0.5 °C to indicate clinically utility (green broken lines).

**Fig. 1:**
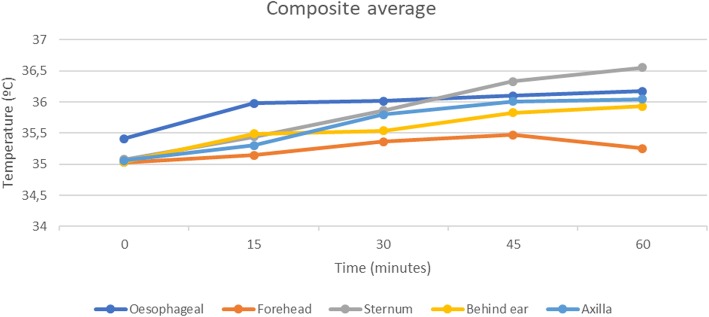
Composite averages of temperature monitors placed on the forehead, sternum, behind the ear, in the axilla and compared to an oesophageal monitor

Figure 1, a composite average of all readings at specific time intervals, does not accurately represent the usefulness of the skin temperature monitors but merely offers a visual representation of the trends of the monitors studied. The oesophageal monitor started at a higher base line temperature when compared to the skin temperature monitors and approximated core temperature within 15 min, this is a function of the relatively rapidly responding oesophageal monitor when compared to the skin monitors. Only the axillary skin temperature appeared to visually approach the trend of the oesophageal temperature monitor. The sternally placed probe overshot the oesophageal temperature readings, while the forehead monitor appeared to initially approximate the trend of the oesophageal monitor but began to under read after 45 min. The monitor behind the ear (over major vessels to the brain) slowly approached that of the oesophageal probe but failed to follow its trend.

The readings from the skin temperature monitors placed behind the ear (over major vessels to the brain) did not fall within the clinically pre-defined limits of agreement (±0.5 °C), the bias is − 0.43 °C but with wide limits of agreement.

**Fig. 2:**
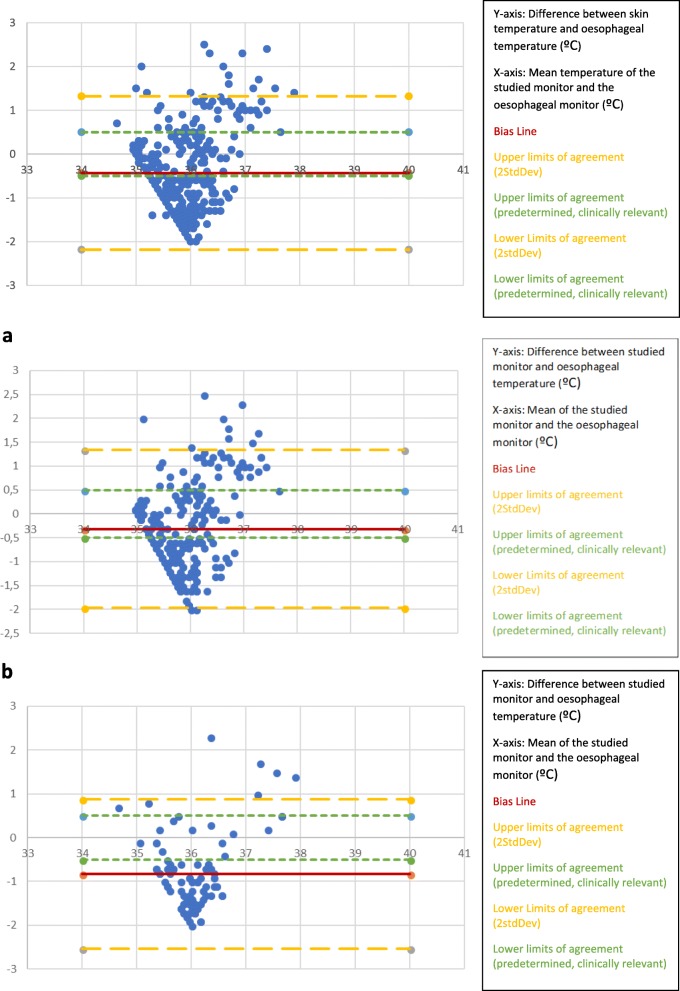
Altman-Bland plots of skin temperature monitor placed behind ear as compared to oesophageal temperature. (*n* = 92; 368 data points). **a** Altman-Bland plots of skin temperature monitor placed behind the ear (over major vessels to the brain) compared to oesophageal temperature WITH a forced air warmer (*n* = 75; 300 data points). **b** Altman-Bland plots of skin temperature monitor placed behind the ear (over major vessels to the brain) compared to oesophageal temperature WITHOUT a forced air warmer (*n* = 17; 68 data points)

To determine the impact of FAW use we created a graph for patients in whom an FAW was used (Fig. 2a) and a graph for patients in whom an FAW was not used (Fig. 2b). After separately analysing data from participants with and without a FAW.

The skin temperature monitors placed behind the ear (over major vessels to the brain) with a FAW did not fall within the clinically pre-defined limits of agreement of − 0.5 and + 0.5 °C. The bias line was − 0.31 °C, the limits of agreement were wide.

The skin temperature monitors placed behind the ear (over major vessels to the brain) without a forced air warmer did not fall within the clinically pre-defined limits of agreement of ±0.5 °C.

The readings from the skin temperature monitors placed in the axillary apex did not fall within the clinically pre-defined limits of agreement of ± − 0.5 °C. The bias is − 0.26 °C and the limits of agreement were wide.

**Fig. 3:**
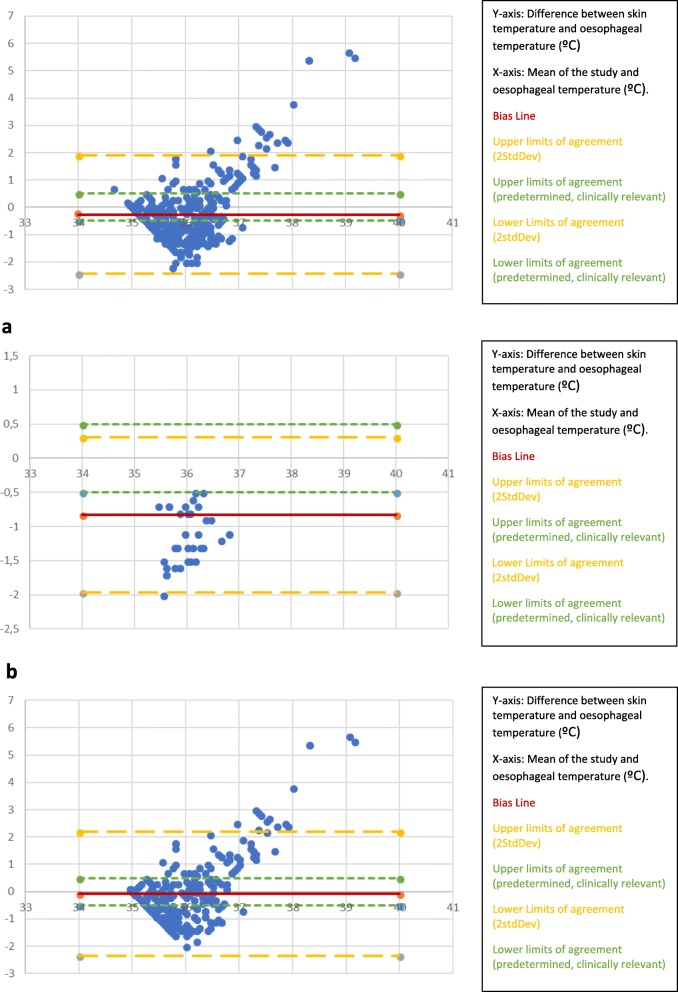
Altman-Bland plots of skin temperature monitor in the axillary apex compared to oesophageal temperature. (*n* = 92; 368 data points). **a** Altman Bland Plots of skin temperature monitor placed in apex of axilla compared to Oesophageal temperature WITHOUT a forced air warmer (*N* = 17; 68data points). **b** Altman Bland Plots of Skin temperature monitor placed in apex of axilla compared to Oesophageal temperature WITH a forced air warmer. (*N* = 75; 300 data points)

To determine the impact of FAW use we created a graph for patients in whom an FAW was used (Fig. 3a) and a graph for patients in whom an FAW was not used (Fig. 3b).

The skin temperature monitors placed in the axilla WITHOUT A FAW did not fall within the clinically pre-defined limits of agreement of ±0.5 °C. All the skin temperature monitor readings under-read the oesophageal temperature.

The skin temperature monitors placed in the axilla WITH A FAW did not fall within the clinically pre-defined limits of agreement of ±0.5 °C. Some skin temperature monitor readings over-read the oesophageal temperature.

The readings from the skin temperature monitors placed over the middle of the sternum did not fall within the clinically pre-defined limits of agreement of ±0.5 °C. The bias line was − 0.66 °C, the limits of agreement were wide.

**Fig. 4:**
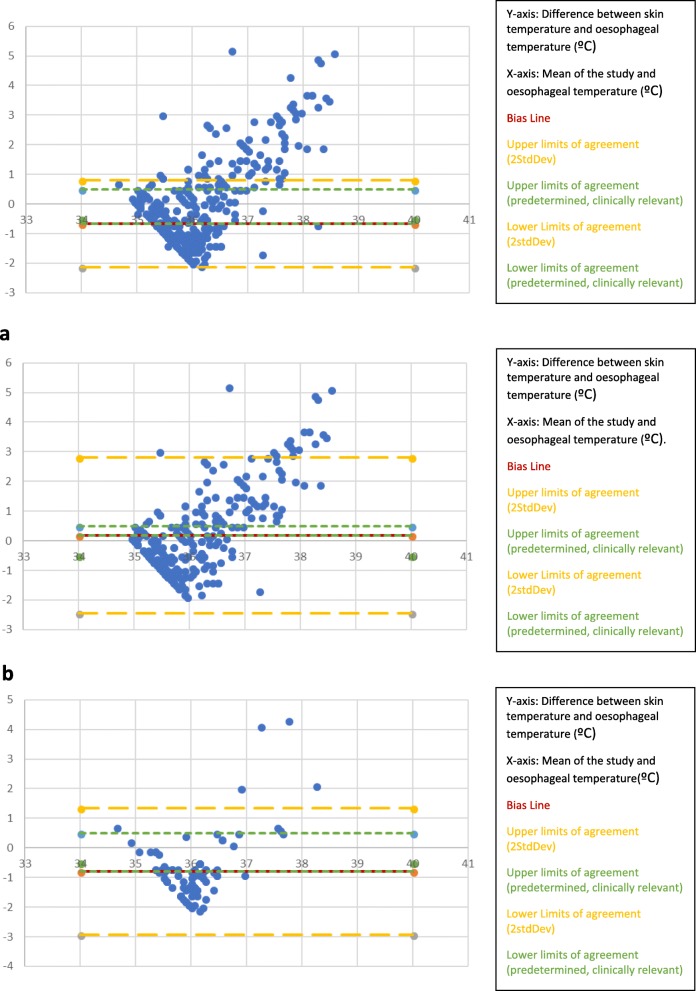
Altman-Bland plots of skin temperature monitor on the middle of the sternum as compared to oesophageal temperature. (*n* = 92; 368 data points). **a** Altman-Bland Plots of skin temperature monitor on the middle of the sternum compared to oesophageal temperature WITH a forced air warmer (*N* = 75 [300 data points]). **b** Altman-Bland Plots of skin temperature monitor on the middle of the sternum compared to oesophageal temperature WITHOUT a forced air warmer (*N* = 17; 68 data points)

This plot demonstrated a significant number of readings over 37.5 °C which may have been caused using an FAW. To explore this, we created a graph for patients in whom an FAW was used (Fig. 4a) and a graph for patients in whom an FAW was not used (Fig. 4b).

This plot demonstrated a number of readings over 37.5 °C which may have been caused by the use of a FAW.

In the patients where a FAW was not used more under-reading was noted.

**Fig. 5:**
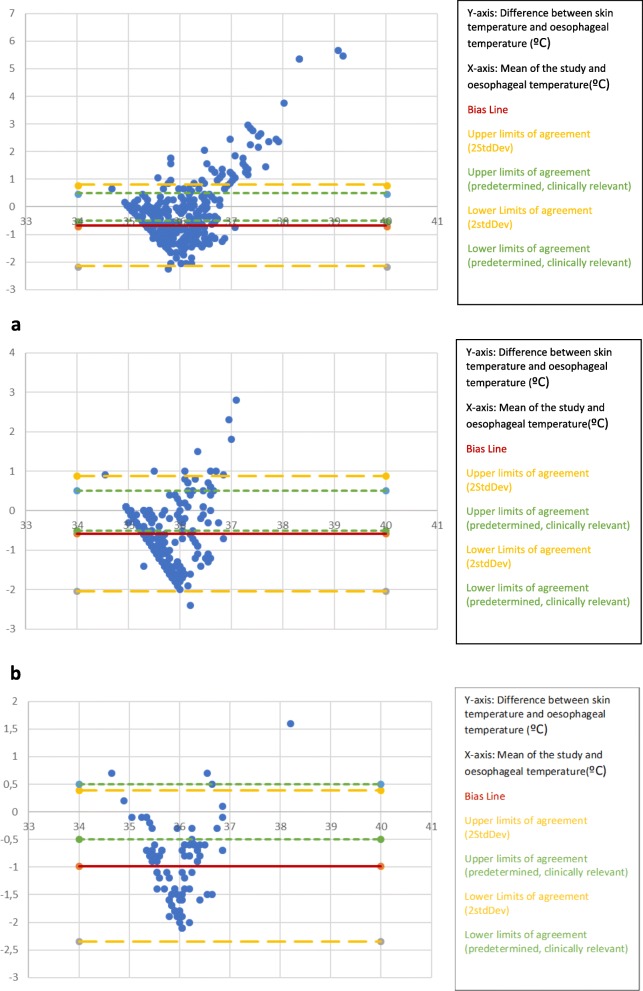
Altman-Bland plots of skin temperature monitor over the middle of the forehead as compared to oesophageal temperature. (*n* = 92; 368 data points). **a** Altman-Bland Plots of skin temperature monitor over the forehead as compared to oesophageal temperature WITH a forced air warmer. (*N* = 75; 300 data points). **b** Altman-Bland Plots of skin temperature monitor over the forehead as compared to oesophageal temperature without a forced air warmer. (*N* = 17; 68 data points)

The readings from the skin temperature monitors placed on the forehead did not fall within the clinically pre-defined limits of agreement of ±0.5 °C. The bias line was − 0.66 °C. This plot demonstrated over-reading. To explore this, we created a graph for patients in whom an FAW was used (Fig. 5a) and a graph for patients in whom an FAW was not used (Fig. 5b).

A FAW does not appear to affect the reading of monitors placed over the forehead.

Surface skin temperature monitoring using the study thermometers (TraxIt®) did not fall within the pre-defined clinical limits of agreement.

Across all the graphs (Figs. 2, 3, 4 and 5) a clustering of data points in a diagonal line in the lower left area of the plot was noted. This most likely resulted from the thermistor temperature probe warming up rapidly to approximate the core temperature as compared to the slow warm up of the skin temperature monitor; this diagonal line acts as the starting/base difference between the two monitor readings. As the skin temperature monitor warms up the data points are spread perpendicular to the diagonal base line. These plots demonstrate under reading at lower temperatures (more negative difference) and over reading at higher temperature (more positive difference).

Feedback from clinicians participating in the data collection bemoaned the difficulty of reading the skin surface monitor; difficulty in accessing these monitors during surgery; as well as the slow response time in “heating up”. There were no complaints from participants and no adverse skin reactions resulting from the placement of the skin surface temperature monitors.

## Discussion

This study aimed to determine if the Traxit temperature monitor (newly available at our institution, cheap, easy to use in many clinical settings, non-invasive and quick to apply) produced readings that agreed with the conventional standard of care sufficiently well to allow substitution in our clinical setting.

This study found that the Traxit thermometer did not perform adequately enough to be used during general anaesthesia. None of the sites tested achieved the clinically relevant predetermined limits of agreement (+/− 0.5 °C).

Our understanding of thermoregulation and anaesthesia has progressed significantly over the last 30 years. Hypothermia is defined as a body temperature reading below 36.0 °C [2]. It is associated with many negative clinical consequences including altered pharmacodynamics and pharmacokinetics, poor wound healing, and patient discomfort with shivering on waking or during awake procedures [2]. Shivering also causes increased oxygen demand and impairs monitoring devices causing artefacts on ECG and NIBP readings. Monitoring for hyperthermia, especially in patients being actively warmed, is as important as monitoring for hypothermia. It assists in detecting pathologies (e.g. malignant hyperthermia) and prevents the increased metabolic demand of hyperpyrexia. Many regions in the world demand the maintenance of normothermia as part of their national guidelines and minimum standards, and some regions, notably in the USA, may withhold full payment if normothermia is not maintained during anaesthesia [9, 10]. The maintenance of normothermia has become a crucial component of providing anaesthesia and an essential part of a patient’s journey through anaesthesia [9].

Body temperature is not homogeneous [2]: the temperature of deep thoracic, abdominal, and central nervous system (considered core temperature) usually range in temperature, the arms and legs are cooler and much of the skin surface is cooler still although also heterogeneously distributed. Unlike core temperature, which is tightly regulated, skin temperature can vary considerably. It is a function of environmental exposure and depends on exposure, central compartment temperature and thermoregulatory vasomotor activity [1]. Due to multiple factors there is no single tissue temperature that can be considered a “gold standard”. Core temperature can be determined by measuring temperature in the nasopharynx, with a pulmonary artery catheter or in the distal oesophagus. Carefully obtained oral, axillary and bladder temperatures have been shown to approximate core temperatures sufficiently for clinical use. Axillary, oral or forehead skin surface temperatures have previously been substituted for oesophageal or nasopharyngeal temperatures during regional anaesthesia [11].

Earlier reviews of non-invasive temperature monitors have shown varied results and provided confusing conclusions. As shown by Hooper and Andrews [11] the results they reported indicated that tympanic monitoring is commonly used but high-quality evidence supporting the accuracy of tympanic thermometry is lacking, and in fact, the most recent high-quality studies evaluating the accuracy of this instrument fail to show support for its use. This is further seen in practice when the measurement of core body temperature, depending on what method is used and where the measurement is made, is subject to considerable error [12]. It is noted in the 2018 update of the NICE guidance on Hypothermia [13] that healthcare professionals should be aware of, and carry out, any adjustments that need to be made in order to obtain an estimate of core temperature from that recorded at the site of measurement and be aware of any such adjustments that are made automatically by the device used. It highlights the inaccuracies of axilla and sublingual readings. It goes on to warn health care professionals of possible inaccuracies in core temperature estimation when using peripheral sites, such as sublingual or axilla, especially in patients whose core temperature is outside the normothermic range. An indirect estimate of core temperature is the reading produced by a thermometer after a correction factor has been applied, this adds a layer of inaccuracy.

Accurately measuring a patient’s temperature remains an inherently essential step in maintaining normothermia. An appropriately placed oesophageal temperature probe has been shown to approximate core body temperature. However, the placement of an oesophageal temperature probe is a semi-invasive technique and not well tolerated by awake patients. To date there are many suitable methods to measure a patient’s temperature when they are awake, however they are not available at our institution. Semi or non-invasive temperature monitors include the SpotOn Thermometer [5] (single-use sensor consists of a thermal insulator adjacent to the skin which is covered by a flex circuit, the flex circuit actively regulates its temperature to create a zone of perfect insulation), DoubleSensor thermometer [6] (which consists of two temperature probes on each side of a standardized insulator, one side adjacent to the patient’s skin and the other facing the environment, the heat coefficient of the insulating material is known and core temperature can be calculated using a proprietary formula) and the Temple Touch Pro [7] (which estimates core temperature from skin over the temporal artery but also requires separate monitor and display unit).

An alternative temperature monitoring device available at our institution is the Traxit® Wearable Children’s Underarm Thermometer. This thermometer utilises liquid crystals that have phase change properties when heated and is read using a dot matrix grid. The purported advantage of such a monitor is that it could be placed on the patient before anaesthesia while in the ward and could serve as the sole temperature monitor throughout the patient’s anaesthetic journey from ward to theatre and back again. Unfortunately, they were shown not to be of clinical value.

This study used Altman-bland plots to compare two methods of measurement, the pharyngeal/oesophageal thermistor (the standard of care at our institution) and the Traxit® Wearable Children’s Underarm Thermometer. Altman-bland plots are considered superior to simple regression/correlation analysis. Previous studies comparing non-invasive temperature monitors used similar methods to analysis performance of monitors. Kimberger et al. [6] collected data from 56 patients (general anaesthesia and regional anaesthesia) and found the average bias to be − 0.13 °C with narrow limits of agreement (− 0.65 to 0.40 °C). The Spoton deep forehead non-invasive temperature monitor was evaluated by Kato [5] in 2015. Kato examined 20 patients following cardiac surgery and compared the Spoton thermometer to the pulmonary artery catheter thermometer, they were able to collect and analyse over 16,000 data points. Altman-Bland plots were again used and clinical limits of agreement were agreed to be +/− 0.5 °C. A bias of − 0.28 °C with narrow limits of agreement were found. In 2017 Evron and colleges [7] examined the Temple Touch Pro device, they also used Altman-bland plots to compare the Temple Touch Pro to reference temperature measured in the naso-pharynx or oesophagus. Evron tested the hypothesis that the Temple touch pro non-invasive temperature measurement system estimates core temperature to within 0.5 °C of reference values. They were able to conclude cutaneous temporal TTP temperatures were sufficiently accurate for routine clinical use, with 94% of all measurements across a range of ages and types of surgery being within ±0.5 °C of reference distal oesophageal or naso-pharyngeal reference core temperatures. This study clearly shows that the Traxit® Wearable Children’s Underarm Thermometer is not of clinical value. Traxit® Wearable Children’s Underarm Thermometer had bias values of − 0.43 °C (behind the ear (over major vessels to the brain)), − 0.26 °C (in the axilla), − 0.66 °C (sternum) and − 0.66 °C (forehead) with wide limits of agreement. None of the results fell within the pre-defined clinical limits of agreement of +/− 0.5 °C.

Importantly, 55 of the 400 (14%) monitors failed to give a reading above 35 °C, the minimum displayed temperature of the Traxit® Wearable Children’s Underarm Thermometer. This was a notable finding when compared to the reliability of the oesophageal temperature monitor which never failed to heat up.

Forced air warming devices (FAW) were placed at the discretion of the individual anaesthetic providers based on their assessment of patient and surgical factors. FAW devices consist of a heat generating device and a fan or blowing system connected to a perforated ‘blanket’ that is placed over the patient. Many devices are available, the Bair Hugger 3 M is available in the intuition that conducted this study. It has three temperature settings and two fan modes. They work by radiant shielding and convection. They provide a buffer of warm air over the patient’s skin so heat loss via radiation is minimised. FAW devices heat the patient’s skin by convection/facilitated conduction by inducing a flow of warm air over the patient’s skin. Ambient temperature should not affect the monitor as they are insulated but the mechanism of FAW devices may impact on the accuracy of the surface skin temperature monitors. This was investigated by separating out the study participants into those where a FAW device was used and those where one was not used. The use of a FAW did not impact the results, in that no monitors provided reading within the pre-determined limits of agreement. The use of a FAW impacted readings by falsely increasing the temperature readings.

A theoretical limitation of skin temperature monitoring is the establishment of a peripheral to core temperature gradient. This gradient develops as the patient vasoconstricts in the presence of hypothermia to preserve heat and maintain core temperature. Skin temperature is often quoted as being up to 2 °C cooler than core temperature. However, the thermoregulatory response to hypothermia is reduced when under general anaesthesia. The threshold for skin vasoconstriction is reduced, and only becomes active at core temperatures less than 34.5 °C [9, 10]. This, in combination with the vasodilatory effects of general anaesthetic agents (propofol, volatile agents and regional techniques), makes interpreting and assigning a definitive number to skin temperatures in relation to core temperatures under anaesthesia difficult. It is also noted that temperature of vital organs is not homogenous, for example the brain may be warmer than the kidneys. Also, skin temperature has a wide variation determined by exposure and proximity to the central components. Big toe temperatures are different to axillary temperatures which in turn are different to forehead temperatures. This study aimed to find a suitable device that could substitute the standard of care in our facility which is the pharyngeal/oesophageal thermistor.

FAW devices may be better at preventing hypothermia than correcting it once hypothermia and vasoconstriction is established. Heating of the skin may not translate into core temperature warming until skin perfusion has improved, vasoconstriction has reversed and the causes of heat loss abated. However, local warming of the skin may improve local circulation even in the presence of central hypothermia [14]. The effect of a FAW are difficult to predict and may serve to only externally warm the monitoring device and not represent a true skin temperature reading.

Further limitations to this study are, the pragmatic sampling used may limit the reproducibility of the study as it resulted in a heterogenous patient and surgical population being studied. Thermodynamics of an obese patient presenting for laparoscopic cholecystectomy may differ as compared to a chronically ill and wasted orthopaedic patient. Further limitations include non-uniformity of ambient operating theatre temperatures, as well as a variation in pharmacotherapy for which data was not collected. This may have influenced the vasodilatory response of patients. Although the principal investigator was present at the start of the procedure, individual participants data was recorded by the attending anaesthetic provider. The Traxit Skin temperature monitor has been designed as a paediatric temperature monitor, the impact of probe size and foot print area on measuring an adult’s temperature is unknown.

Strengths of this study are its prospective nature and pragmatic study design. The large number of data points has allowed sub-analysis of study groups and has yielded meaningful results.

Future research is required to find the most suitable location and device for temperature monitoring in an awake patient where the availability of non-invasive temperature monitors is limited. Temperature monitoring should be compulsory and be emphasized as an important function of the anaesthetic provider.

## Conclusion

Temperature measurements recorded with a TraxIt® Wearable Children’s Underarm Thermometer (liquid crystal phase change dot matrix thermometer) did not fall within the clinically applicable predefined limits of agreement. They also demonstrated a high failure rate and were found to be poorly responsive. They proved to be unreliable, unresponsive and inaccurate. The practicality of using a TraxIt® Wearable Children’s Underarm Thermometer is limited. Placement of a TraxIt® Wearable Children’s Underarm Thermometer is not suitable for temperature monitoring during general anaesthesia. Future research is required to find the most suitable location and device for temperature monitoring in an awake patient where the availability of non-invasive temperature monitors is limited.

## Supplementary information

**Additional file 1.** Data Collection Tool.

## Data Availability

The datasets used and analysed during this study are available from the corresponding author Dr. G Simpson, drgcsimpson@gmail.com.
