# Supplementary material for: A prospective observational study testing liquid crystal phase change type thermometer placed on skin against oesophageal/pharyngeal placed thermometers in participants undergoing general anesthesia
Source: BMC Anesthesiol. 2019 Nov 9;19:206. doi: 10.1186/s12871-019-0881-9 (PMC6842509; doi:10.1186/s12871-019-0881-9)
Supplement: Supplementary file 1 — Additional file 1. Data Collection Tool. [file 12871_2019_881_MOESM1_ESM.docx]

**Additional file 1: Data Collection Tool**
